# Small cell oesophageal carcinoma: an institutional experience and review of the literature

**DOI:** 10.1038/sj.bjc.6603611

**Published:** 2007-02-13

**Authors:** E Hudson, J Powell, S Mukherjee, T D L Crosby, A E Brewster, T S Maughan, H Bailey, J F Lester

**Affiliations:** 1 Velindre Hospital, Velindre Road, Whitchurch, Cardiff, CF14 2TL, UK

**Keywords:** small cell, oesophagus, chemotherapy, radiotherapy

## Abstract

Primary small cell oesophageal carcinoma (SCOC) is rare, prognosis is poor and there is no established optimum treatment strategy. It shares many clinicopathologic features with small cell carcinoma of the lung; therefore, a similar staging and treatment strategy was adopted. Sixteen cases referred to Velindre hospital between 1998 and 2005 were identified. Patients received platinum-based combination chemotherapy if appropriate. Those with limited disease (LD) received radical radiotherapy (RT) to all sites of disease on completion of chemotherapy. Median survival of all patients was 13.2 months. Median survival of patients with LD was significantly longer than those with extensive disease (24.4 *vs* 9.1 months, *P*=0.034). This is one of the largest single institution series in the world literature. Combined modality therapy using platinum-based combination chemotherapy and radical RT may allow a nonsurgical approach to management, avoiding the morbidity of oesophagectomy. Prophylactic cranial irradiation is controversial, and should be discussed on an individual basis.

Primary small cell oesophageal carcinoma (SCOC) was first described in 1952 by McKeown (1952), who reported two cases at post mortem examination . It is a rare condition, accounting for between 0.5 and 2.4% of all primary oesophageal malignancies (Casas *et al*, 1997). Prognosis is poor and metastases are often present at the time of diagnosis; patients presenting with disease outside the oesophagus have a median survival of only 1 month without treatment (Casas *et al*, 1997). Owing to its rarity, an optimum treatment strategy has not been established, and various combinations of surgery, radiotherapy (RT), and chemotherapy have been described in the literature.

Small cell lung cancer (SCLC) is a far more common disease, accounting for up to 20% of all primary lung cancers and it shares many clinicopathologic features with SCOC. There is good evidence that combination chemotherapy significantly increases median survival compared to no systemic therapy or single agent chemotherapy in SCLC (Girling, 1996; Agra *et al*, 2003). In addition, in SCLC patients with limited disease (LD) and a complete response to chemotherapy, consolidation thoracic irradiation and prophylactic cranial irradiation (PCI) both increase 3-year absolute survival by 5.4% (Pignon *et al*, 1992; Auperin *et al*, 1999). Therefore, a similar staging and treatment strategy for SCOC was adopted. We report our clinical experience using this strategy and review the published literature on SCOC.

## MATERIALS AND METHODS

All cases of SCOC referred to Velindre Cancer Centre, Southeast Wales, between 1998 and 2005, were identified using an electronic database holding information on all treated cancer patients (Information System for Clinical Organisations, ISCO). Data were collected retrospectively from ISCO and by case note review on demographic details, performance status (PS), treatment, and response to treatment. All patients were required to have a histological diagnosis of small cell carcinoma according to WHO criteria (WHO, 1982). Survival was recorded from the date of histological diagnosis. Staging was analogous to that used in SCLC as proposed by the Veterans’ Administration Lung Group; LD was defined as a tumour mass contained within the oesophagus or perioesophageal tissues, with or without regional lymph node involvement, such that the disease can be encompassed within a tolerable radiation therapy port, and extensive disease (ED) included all other patients (Zelen, 1973). For the literature review, the electronic databases MEDLINE, EMBASE, and Cancerlit were searched using the key words oesophagus, oesophagus, small cell, neuroendocrine, and gastrointestinal. Hand searching of journals, relevant books, and review articles was also carried out. Survival was calculated using the Kaplan–Meier method.

## RESULTS

Sixteen patients were identified, and their clinical details are summarised in Table 1. The age range of all patients was 48–81 years with a median age of 68.5 years and 12/16 (75%) patients were female. Histological diagnosis was established by oesophagogastroduodenoscopy and biopsy in all patients. In all cases, pure SCOC was reported with no other histological subtypes identified. In 8/16 (50%) patients selected, immunohistochemical staining was performed to confirm the diagnosis. Neurone-specific enolase was positive in three patients and CD56 in three patients, supporting the diagnosis of small cell carcinoma. In two patients immunohistochemistry was unhelpful, but a second opinion on the cellular morphology supported the diagnosis of small cell carcinoma. In 8/16 (50%) patients the diagnosis was made on the classical histological appearance of small cell carcinoma and immunohistochemistry was not deemed necessary. In total, 13/16 (81%) patients were staged with a CT scan of the chest and abdomen. Two patients had abdominal ultrasound (Patients 9 and 14) and one patient was deemed too frail for staging investigations (Patient 16). Only one patient (Patient 11) had a bone scan as part of the initial staging investigations. A CT scan of the head was not performed in any patient.

Performance status was recorded in 15/16 (93%) patients. In total, 10/16 patients had a PS of 0–1, 4/16 had a PS of 2 and 1/16 had a PS of 4. In total, 13/16 patients had disease in the lower-third of the oesophagus and 3/16 had disease in the middle-third. No tumours were identified in the upper-third of the oesophagus.

In total, 6/16 (38%) patients had LD, and 9/16 (56%) had ED. Stage was not recorded in 1/16 (6%) patient.

Of those with ED, 7/9 (78%) patients had liver metastases at the time of diagnosis. In total, 6/9 (67%) patients with ED were given chemotherapy; all received four to six cycles of PE (carboplatin AUC 5–6 intravenously (i.v.) in combination with oral etoposide 3-weekly). Patients 13 and 15 received palliative RT to the oesophagus following a partial response (PR) to chemotherapy. Three patients (patients 9, 10, and 14) received no chemotherapy because of poor PS, and were managed with best supportive care. Patient 16 (unknown stage) was treated with palliative RT only.

Five of the six patients with LD were given chemotherapy. Patient 3 received six cycles of ICE (carboplatin AUC 6 i.v. ifosfamide 3 gm^−2^ i.v. and oral etoposide 4-weekly). The other patients received four to six cycles of either PE or CE (cisplatin 60–80 mgm^−2^ i.v. and oral etoposide 3-weekly). All five patients received RT to the oesophagus given with curative intent following a PR to chemotherapy. Patients 3 and 4 also received PCI. One patient with LD (Patient 2) was treated at another cancer centre with radical RT alone. His subsequent treatment was carried out at Velindre hospital.

**Figure 1:**
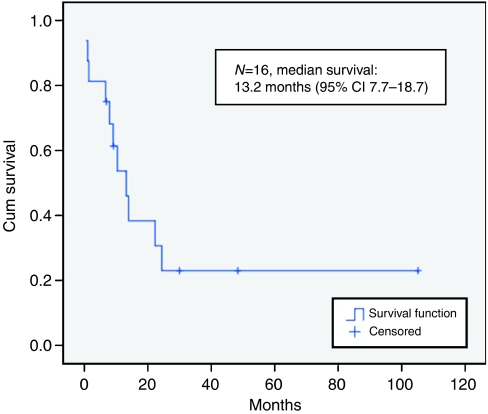
Overall survival of all patients with SCC oesophagus.

Radiotherapy to the oesophagus was given 3–4 weeks after the last cycle of chemotherapy. The dose given was 30–50 Gy in 12–25 fractions over 2–5 weeks using megavoltage photons. Treatment was 2D or 3D-planned to cover all areas of disease including any involved lymph nodes. When used, PCI was given concurrently with RT to the oesophagus. The median survival of all 16 patients was 13.2 months (95% CI 7.7–18.7), and is shown in Figure 1.

To date, 7/9 (78%) patients with ED have died. Patients 12 and 15 are alive 30 and 7 months after diagnosis, respectively. The median survival of all patients with ED is 9.1 months (95%CI 3.3–14.8).

**Figure 2:**
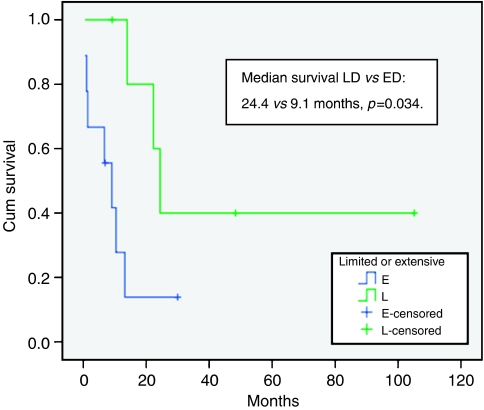
Overall survival of patients with limited *vs* ED.

Of the six patients with LD, patients 1 and 3 are alive with no evidence of recurrent disease, 104 and 48 months after diagnosis respectively. Patient 5 is alive 9 months after diagnosis but has relapsed locally. Patient 2 relapsed locally and died 19 months after diagnosis. Patient 4 developed malignant ascites and patient 6 bone metastases, dying 24 and 14 months after diagnosis, respectively. The median survival of all patients with LD was 24.4 months (95% CI 19.9–28.9), which was significantly longer than those with ED (*P*=0.034; Figure 2).

## DISCUSSION

Primary SCOC is a relatively rare condition, and we have reported one of the largest single-institution series in world literature. The largest retrospective analysis has been carried out by Casas *et al* (1997), who analysed data on 199 evaluable patients found in the literature. They reported a male / female ratio of 1.57 : 1, with 95% of tumours situated in the mid- and lower oesophagus. One-third of cases were mixed tumours with squamous differentiation also identified in addition to small sell carcinoma. Our series has a male /  female ratio of 1 : 4, which is at variance with all other published literature on SCOC. The relatively small sample size may in part explain the difference, but a recent increase in the proportion of female smokers may also play a role. We identified no tumours in the upper-third of the oesophagus, concurring with Casas *et al*, (1997). All tumours were pure small cell carcinoma with no mixed tumours reported. However, none of our patients had surgery, and the absence of other histological subtypes may simply reflect the small size of the tissue samples examined at the time of diagnosis. More than half of patients in our series had metastatic disease at diagnosis, as in most other published series (Casas *et al*, 1997). This is similar to SCLC, indicating SCOC is an aggressive, systemic disease. The rarity of SCOC and absence of randomised trials means the optimum treatment strategy remains unclear.

For the treatment of LD with curative intent, RT alone has been used; however, results generally have been disappointing and long-term survivors are only rarely reported (Doherty *et al*, 1984; Huncharek *et al*, 1995; Medgysey *et al*, 2000). The only patient in our series treated with radical RT alone died 19 months after diagnosis.

There are few reports of surgery being used as the sole treatment modality and, as with RT, most institutional series report a poor outcome with surgery alone. Hosokawa *et al* (2005) reported on five LD patients treated with radical oesophagectomy alone, and only one patient survived more than 24 months. Craig *et al* (1995) reported on five patients treated with surgery alone, and only one survived more than 12 months. Law *et al* (1994) treated three patients with radical oesophagectomy with a median survival of 11.6 months.

The role of chemotherapy in SCLC is well established (Agra *et al*, 2005; Girling, 1996). Evidence suggests that extrapulmonary small cell carcinoma is also a chemosensitive disease (Lester *et al*, 2006; Levenson *et al*, 1981).There is a general consensus in the recent literature that systemic chemotherapy should also be used in the treatment of SCOC. Casas *et al* (1997) reported a median survival of 20 months in those patients receiving systemic chemotherapy compared to only 5 months in those having local treatment only. Performance status was poorly reported in the series included in the Casas review; good PS is a better prognostic factor in SCLC (Bremnes *et al*, 2003), and it is reasonable to assume it is so in SCC. Poor PS patients tend not be given chemotherapy, and the longer survival in the chemotherapy group may therefore be owing in part to the better PS of these patients. As a result of relatively poor outcomes using local treatment only and the apparent chemosensitivity of SCOC, combined modality treatment strategies were developed. The MD Anderson Cancer Center reported on four LD patients treated with chemotherapy followed by RT and/or oesophagectomy. One patient remains alive and disease–free, 57 months after diagnosis (Medgysey *et al*, 2000). Craig *et al* (1995) reported a patient surviving 96 months after oesophagectomy and adjuvant chemotherapy. In our series, of the six patients with LD, two are alive with no evidence of recurrent disease 47 and 79 months after diagnosis, respectively. Both received chemotherapy followed by RT to all sites of disease.

Patients with LD were offered PCI at the discretion of the treating oncologist. PCI in SCLC patients with LD has been shown to decrease the incidence of brain metastases from 59 to 33%, and increase 3-year absolute survival by 5.4%. The incidence of brain metastases in SCOC is not known, and to our knowledge, no study has been reported on the use of PCI in SCOC. To date, none of the patients with LD has relapsed in the brain and only two patients had PCI.

## CONCLUSION

Caution should be applied in basing treatment strategies on retrospective data, as poor record keeping and bias may influence results. However, for localised disease it does seem that combined modality therapy using platinum-based combination chemotherapy and RT to the oesophagus may provide effective local control and avoid the morbidity of extensive surgery. Oesophagectomy or RT alone are probably inadequate, and if used, should be combined with adjuvant or neoadjuvant platinum-based chemotherapy. The role of PCI is controversial, and should be discussed with patients on an individual basis. For those patients presenting with metastases, palliative chemotherapy may prolong survival, and RT can be used for local control in selected good PS patients.

## Figures and Tables

**Table 1:** Clinical and treatment details of the 16 SCC oesophagus patients

**Patient**	**Age**	**Sex**	**PS**	**Stage**	**Primary Site**	**Site of metastases**	**Chemo (cycles)**	**Response**	**Oesophageal RT**	**PCI**	**Relapse site**	**Survival (months)**
1	77	F	0	LD	Lower		4 PE	PR	40 Gy/15F			104, alive
2	71	M	1	LD	Lower				44 Gy/12F		Local, nodal	19
3	59	M	0	LD	Lower		6 ICE	PR	40 Gy/15F	30 Gy/10F		48, alive
4	48	F	0	LD	Lower		6 CE	PR	50 Gy/25F	30 Gy/10F	Ascites	24
5	51	F	0	LD	Mid		4 PE	PR	45 Gy/25F		Local	9, alive
6	50	F	0	LD	Mid		4 PE	PR	50 Gy/25F	30 Gy/10F	Bone	14
7	53	F	UK	ED	Lower	Abdo nodes	4 PE	PR	30 Gy/15F		Local	12
8	67	F	2	ED	Lower	Liver, spleen	4 PE	UK			UK	6
9	72	F	4	ED	Lower	liver						0.5
10	73	F	2	ED	Lower	Liver						1
11	71	M	1	ED	Lower	Liver	4 PE	PR			PD	10
12	72	F	1	ED	Mid	Liver, lungs, SCF	6 PE	PR			PD	30, alive
												
13	71	F	1	ED	Lower	Liver	4 PE	PR	25 Gy/10F		Local, liver, brain	13
14	65	F	2	ED	Lower	Liver					PD	2
15	58	M	1	ED	Lower	Abdo nodes, pancreas	4 PE	PR	40 Gy/15F			7, alive
16	81	F	2	UK	Lower	UK			8 Gy/1F		UK	7

Abdo=abdominal; CE=cisplatin and etoposide; DF=disease-free; ED=extensive disease; F=female; ICE=ifosphamide, cisplatin and etoposide; LD=limited disease; M=male; PE=carboplatin and etoposide; PCI=prophylactic cranial irradiation; PS=performance status; PR=partial response; RT=radiotherapy; SCF=supraclavicular fossa; UK, unknown
